# Supplementary material for: Learning important common data elements from shared study data: The All of Us program analysis
Source: PLoS One. 2023 Jul 7;18(7):e0283601. doi: 10.1371/journal.pone.0283601 (PMC10328251; doi:10.1371/journal.pone.0283601)
Supplement: Attachment — Submitted filename: Response to Reviewers.docx [file pone.0283601.s001.docx]

Dear Dr. Jake Michael Pry,

We thank you and the reviewers for the input and constructive comments on our manuscript. We have thoroughly reviewed each comment and our manuscript and have edited our paper taking into account each comment. We are now submitting the edited and improved version of our manuscript.

See below our response (in bold font) to each reviewer comment. Our response follows the reviewer’s comments (shown in regular un-bolded text).

Thank you,

Craig Mayer and Vojtech Huser

Reviewer #1

This publication is in a combination of present and past tense. Even though the All of US program maybe continuing to enroll participants, the data for this study was obtained at a specific timeline (late 2021), and the analyses performed retrospectively, and hence could be described in past tense.

**Thank you for the comment. We have changed the portions where we discuss our analysis, results and conclusions, to conform to past tense. For the purposes of describing the All of Us program and their data collection practices we maintain the text in present tense since we are describing the ongoing methodology of the program itself. While our analysis and the results we found were done in the past and therefore in the past tense, the All of Us program is ongoing, and we feel should be described in present tense for that reason.**

Reviewer #2

1) Several typos and grammar errors. Please correct.

**Thank you for your comments. We have corrected typos and grammar mistakes throughout the manuscript.**

2) Abstract:
Abbreviation PPI not defined.

**We have defined PPI in the Abstract where the acronym first appears.**

“originated from a previous data collection initiative, such as PhenX (17 CDEs) and PROMIS (15 CDEs)” plr: data collection initiatives

**We have made the term data collection initiative plural in the text of the Abstract to conform with the multiple initiatives discussed.**

3) 2.1 All of Us program
Missing is the aim of the initiative and the planed follow-up. Is biomaterial collected by AoU? Is genetic data available? Please add.

**We added the aims and a brief description of additional data types. However, it would be out of scope to further discuss these data types and process as the manuscript is focused on CRF and EHR data and the presence of common data elements. The addition of these data types beyond just mentioning them do not contribute to the conclusions as they are not part of the common data model structure. The further details regarding the All of Us program was added to this section (2.1).**

4) The structures of the data elements and values are rather complex and not easy to grasp. Add figures which present the data structures and hierarchies and also add examples in the text.

**Figure 1 along with an explanation was added to section 3.2 to clarify this process and structure.**

5) Table 2: Why is the column “CRF” blank for some elements? Explain in legend to table 2.

**The reason the CRF column is blank for some data elements is that these elements originate from a research visit instead of a CRF. This explanation was added as a footer to Table 2.**

6) Element ‘Current State’: Please explain what that means.

**To avoid initial confusion about the meaning of this data element, it was removed from Table 2. When it is mentioned later in the text in section 4.1.1, we explain that ‘Current State’ refers to the geographic location of the participant.**

7) l 233: GROR: It is not explained what that is.

**We added an explanation of what GROR refers to in section4.1.2. The exact meaning of the GROR acronym is not made apparent by the All of Us program or OMOP terminology. Instead, we explained the general purpose of the GROR CRF.**

8) l 245: Brackets not consistent.

**We fixed this with the addition of a close bracket for bracket agreement.**

9) Table 6: Add the event counts. Why do the “Percent of Element Values” not add up to 100%? Please explain what the percent refers to. Please additionally use such a reference population that the percentages add up to 100%.

**Table 6 has been redone to include the count of total responses and responses by value chosen to describe the percentage. All values were included to see 100% of the population’s responses. A sentence was added before the table to explain the contents.**

10) 5.1 EHR and research data integration:
“An example of this can be seen with cigarette smoking quantification (OMOP concept_id: 40766929) which may be established by research questionnaire (in 97.2% of instances) but later updated via EHR import (in 2.8% of instances).”
Discuss the possibility of bias because of the low follow-up percentages which might result in selective, biased information.

**Sentences were added to section 5.1 to discuss this possibility. We have added sentences to this section describing the consideration of bias in the data due to the differences in population follow-up caused by the inclusion of EHR data instead of research process repetition.**

11) l. 388f: grammar error

**This sentence was edited to ensure proper grammar and clarify meaning.**

12) 5.3 Types of CDEs:
Please illustrate the two axes and the number of elements contained in the four sets by a table or Venn diagram.

**Table 9 was added to the section to better show this crossover and process. This should help clarify the premise described regarding the types of CDEs and the axes.**
